# Comparison of Methods to Assess Discretionary Salt Intake among Nonpregnant Women of Reproductive Age in Punjab, India

**DOI:** 10.1016/j.cdnut.2025.107456

**Published:** 2025-05-05

**Authors:** Yvonne E Goh, Mari S Manger, Mona Duggal, Reena Das, Surbhi Agarwal, Shipra Saklani, Deepmala Budhija, Manu Jamwal, Bidhi L Singh, Julie M Long, Jamie Westcott, Charles D Arnold, Nancy F Krebs, Rosalind S Gibson, Kenneth H Brown, Christine M McDonald

**Affiliations:** 1 Division of Gastroenterology, Hepatology and Nutrition, Department of Pediatrics, University of California, San Francisco, San Francisco, CA, United States; 2 International Zinc Nutrition Consultative Group; 3 Post Graduate Institute of Medical Education & Research, Chandigarh, India; 4 Department of Pediatrics—Section of Nutrition, University of Colorado School of Medicine, Aurora, CO, United States; 5 Department of Nutrition and Institute for Global Nutrition, University of California, Davis, Davis, CA, United States; 6 Department of Human Nutrition, University of Otago, Dunedin, New Zealand

**Keywords:** discretionary salt intake, salt intake, sodium intake, dietary assessment, nonpregnant females of reproductive age, fortification, India

## Abstract

**Background:**

Accurate and precise estimates of discretionary salt intake are critical for the design of salt fortification programs and salt reduction interventions.

**Objectives:**

This study aimed to compare 4 methods of estimating discretionary salt intake among nonpregnant females of reproductive age in Punjab, India.

**Methods:**

One-day, observer-recorded, weighed food records (WFRs), household salt disappearance (HHSD) data, duplicate diet composites, and samples of household salt and milk were collected from 100 females and repeated in a subset of 40 to adjust for intraperson variation and estimate usual discretionary salt intake. Diet composites were also replicated from 40 randomly selected WFR but prepared without the addition of discretionary salt. The duplicate diet composites’ sodium and iodine contents were analyzed using inductively coupled plasma (ICP)-optical emission spectrometry and ICP-mass spectrometry, respectively. The iodine content of household salt samples was analyzed using the ion-selective electrode method. The association and agreement between the WFR method, the selected reference method, and the HHSD, replicate diet (RD), and iodine methods (IMs) were explored using correlation and Bland–Altman analyses.

**Results:**

Mean ± standard deviation (SD) discretionary salt intakes according to the WFR, HHSD, RD, and IM methods were 4.7 ± 1.8 g/d, 5.7 ± 2.6 g/d, 4.1 ± 2.1 g/d, and 7.8 ± 5.3 g/d, respectively. The RD method showed the strongest correlation (*ρ* = 0.76; *P* < 0.001) and the smallest mean difference ± SD (−0.68 ± 1.25 g/d), with limits of agreement from −3.18 to 1.82 g/d, compared with the WFR method. However, the HHSD method was also moderately correlated (*ρ* = 0.48; *P* < 0.001) and showed good agreement [0.98 ± 2.12 (−3.27, 5.23) g/d] with the WFR despite lower precision.

**Conclusions:**

Although intensive to implement, the WFR and RD methods produce precise estimates of discretionary salt intake. Repeated measurements may improve the precision of the HHSD method for large population-based surveys.

## Introduction

The reduction in the prevalence of iodine deficiency disorders via universal salt iodization represents a major public health achievement [1]. Recent technological innovations have also enabled salt to be fortified with micronutrients beyond iodine [2,3]. Therefore, in settings where micronutrient deficiencies are prevalent, consumption of fortifiable staple foods is low or variable, and coverage of iodized salt is high, salt fortification with additional essential micronutrients may represent a cost-effective strategy to improve micronutrient intake and status. On the other hand, there are ongoing efforts to reduce salt intake globally to control and prevent hypertension and related noncommunicable diseases [4,5]. Accurate and precise estimates of discretionary salt intake are crucial for the evidence-informed design of both salt fortification interventions and sodium reduction efforts.

Discretionary salt refers to salt that is directly added to recipes during preparation or to foods before consumption. Discretionary salt is distinct from total sodium, which also encompasses sodium that is intrinsic in certain foods and contributed by processed/preprepared foods, snacks, spice mixtures, etc. Assessing total sodium intake using the reference standard 24-h urinary sodium excretion method is challenging under field conditions; however, estimating discretionary salt intake, a fraction of total salt intake, is more so because of the technicalities involved in differentiating discretionary salt from total salt consumption [6]. The lithium-marker technique involves labeling discretionary salt with lithium and measuring its excretion in 24-h urine samples at baseline and ≥3 more time points after the ingestion of the lithium-labeled salt [7]. Lithium is not metabolized by the body, and therefore, the excreted lithium tracer in urine is used as an indicator of discretionary salt intake. Although some consider this method as the reference standard, the time and resources that are required to implement it make it unsuitable for use in resource-constrained, field-based studies.

Estimates of discretionary salt intake vary widely across settings and depend on factors such as geographic location, season, specific cooking and food preparation habits [8]. This wide variation across populations underscores the need for accurate, precise, context-specific estimates to inform the design and to monitor the effectiveness of salt fortification programs and salt reduction interventions. There is currently no accepted standardized method for measuring discretionary salt intake in field settings; therefore, comparative studies are needed to help guide researchers on the choice of method to use for their specific purposes.

The objective of this study was to compare 4 different methods for assessing discretionary salt intake among nonpregnant females of reproductive age in Punjab, India. Specifically, we obtained estimates of discretionary salt intake via the following: *1*) collection of 1-d, observer-recorded, weighed food records (WFRs); *2*) 1-d household salt disappearance (HHSD) studies; *3*) the preparation of replicate diet (RD) composites for laboratory analysis of sodium content; and *4*) the iodine method (IM), as described next, and examined measures of association and agreement between the WFR method (as the reference method) and other methods.

## Methods

### Study setting

This study included a cross-sectional sample of 100 nonpregnant females of reproductive age in the Mohali district of Punjab, India who participated in the formative phase of research that was conducted to inform the design of a subsequent randomized controlled trial of multiply-fortified salt [9]. Mohali is an agricultural district that covers a land area of 1098 km^2^ with 383 villages with good access to clean water and electricity [10]. The district has a high literacy rate of 83%. Approximately 56% of females and 57% of males have >10 y of schooling. Most households (95%) in the district use iodized salt [11].

### Study participants

The sampling procedure for the study participants has been previously described [9]. Briefly, a census of all households was carried out in 11 villages in the district. Proportional allocation sampling was then used to select potential participants from each village’s list of eligible households. Potential participants were screened and those who met the following eligibility criteria were enrolled into the study: *1*) 18–49 y of age at the time of enrollment; *2*) not currently pregnant (self-reported), *3*) not severely anemic (defined as a hemoglobin concentration <8.0 g/dL); *4*) permanent resident of the study village with no plans to move or travel outside the village for >4 wk over the next 12 mo, *5*) without serious health problems that required regular visits to a health facility, and *6*) using refined salt as their primary source of household discretionary salt. Signed informed consent was obtained from all females who expressed an interest in participating in the study and met all eligibility criteria. Participants were free to withdraw from the study at any time. Ethical approval for the study was obtained from the Institutional Review Boards of the University of California, San Francisco, United States, and the Post Graduate Institute of Medical Education and Research, Chandigarh, India.

### Data collection

At the time of enrollment, sociodemographic data were collected from all participants including information on salt purchasing and utilization practices. Biochemical and anthropometric assessments were performed within the following 2–3 d. Then, females were scheduled for a comprehensive, full day, in-home dietary assessment that involved collection of the following: *1*) 1-d WFRs capturing the study participant’s dietary intake; *2*) 1-d household-level salt disappearance data; and *3*) simultaneous duplicate diet composite collection. Household salt samples (80 g) were also taken from each participant. These assessments were then repeated in 40 participants on a nonconsecutive day to enable the adjustment of intraperson variation and estimation of usual discretionary salt intake. Females were provided with a stipend to reimburse the cost of the duplicate diet composites after the assessments were completed. In addition, 40 WFRs were selected randomly from which RD composites were prepared in the study kitchen, but without the addition of discretionary salt.

### Comprehensive 1-d dietary assessment

On arriving at the participant’s home early in the morning (06:30–07:00), a female research assistant calibrated the dietary scale (Atom, A123) with a known weight. The participant was then guided to transfer their household salt to a new, preweighed container with a lid provided by the study. This container was measured to the nearest 0.1 g at the beginning of the assessment day before any meals were cooked and at the end of the last meal of the day.

To collect the WFRs, research assistants weighed all foods and beverages, including water, consumed by the participant [12]. All dishes that were cooked while the research assistant was at the participant’s home were carefully documented by weighing all ingredients used and the final cooked weight of the preparation. During the collection of the WFRs, any condiment, including discretionary salt, that was added to recipes during cooking and to food at the table was recorded on dietary scales with a precision of 0.1 g. Because detailed recipes were recorded, the proportion eaten by the female and its corresponding individual ingredients, including salt, could be calculated. In cases where the recipe was prepared on the previous day, the mean of similar recipes collected from all participants was used to calculate the proportion of individual ingredients consumed, including salt.

Duplicate diet composites were collected in parallel with the WFRs [12]. Research assistants collected a duplicate portion of every food and beverage, including water, consumed by the participant. These foods and beverages were collected in large polyethylene plastic containers with airtight lids that were doubly lined with Ziploc bags. 1000 mL water bottles were used to collect any water drunk by study participants. The containers were stored in coolers that were maintained at 4°C or colder using ice packs. An 80 g sample of household salt was taken from every participant, and a 150 mL sample of milk (determined to be a source of iodine because of cleaning practices and products used with cows and buffalos in the study area) was taken for the analysis of iodine only if the participant consumed milk during the assessment. After the last meal of the day was consumed, the research assistant transported all samples and composites to the field laboratory for storage. Before leaving the household, the research assistant provided graduated jugs to participants to record their water intake at night. They were contacted by telephone the next day to confirm the amount of water they drank during the night.

### Processing of duplicate diet composites

The duplicate diet composites were transferred to preweighed empty glass blenders that had been hand-washed with detergent and rinsed with water (Bisleri brand). The duplicate diet composites were then homogenized slowly using a blender, and 40 mL aliquots were transferred to 50 mL conical tubes (Thermo Scientific™) and stored in a −20^°^C freezer in the field laboratory for later transportation to the analytical laboratory.

### Preparation of RD composites

Additionally, a sample of 40 WFRs was randomly selected from which RD composites were prepared by the research assistants in the study kitchen without the addition of discretionary salt. Food ingredients to prepare the dishes were bought in markets in the study area, and research assistants were familiar with the local food preparation methods. The RD composites were also homogenized in blenders, and 40 mL aliquots were taken and stored in −20^°^C freezers for later analysis.

### Analytical determination of sodium and iodine in food samples

Aliquots of the duplicate and RD composites, and samples of water, milk, and refined salt collected during the WFRs were transported by car to the National Institute of Food Technology, Entrepreneurship and Management, Haryana, India, for the analysis of sodium and/or iodine. Sodium concentrations in the duplicate diet and RD aliquots were measured in duplicate using acid digestion and inductively coupled plasma-optical emission spectrometry (Shimadzu Corporation) [13]. The concentration of iodine in the duplicate diet and RD aliquots was determined in duplicate by using hot block extraction and tetramethyl-ammonium hydroxide digestion followed by inductively coupled plasma-mass spectrometry (Sigma Aldrich) [14]. Milk samples were pooled by the participant’s village and type of milk-producing ruminant (cow or buffalo). Iodine concentrations in salt, pooled milk samples, and water samples were analyzed in duplicate using the ion-selective electrode method (Agilent Technologies) [15].

### Estimation of discretionary salt intake

Four methods were used to estimate discretionary salt intake based on the comprehensive dietary assessment.

#### WFR method

The total sum of discretionary salt intake contained in portions of recipes consumed by the participant and added to dishes/fruit/salad before consumption was summed for each participant [12]. This method was chosen to be the reference method for the current study because it involved the direct measurement of discretionary salt used, was measured in real-time by the research assistants, and did not involve any forms of estimation or laboratory analysis that could contribute to other sources of error.

#### HHSD method

The difference in the weight of the salt container at the end of the dietary assessment day and the beginning of the dietary assessment day represented the household’s total discretionary salt use for that day. This value was then divided by the total number of household members to provide a crude estimate of each participant female’s daily discretionary salt intake [16,17].

#### RD method

The sodium content of the original duplicate diet composites and the RD composite aliquots without discretionary salt was determined analytically, as described previously. The analytical values of sodium in the original duplicate diet and RD composite aliquots were multiplied by the weight of the final homogenates (g) to obtain the total sodium content of the duplicate diet and RD composites. The sodium content of the RD composite without discretionary salt was then subtracted from the sodium content of the original duplicate diet with salt to obtain the discretionary sodium content of the diet. This discretionary sodium content was then multiplied by the molar mass of NaCl (2.5413) to provide an estimate of discretionary salt intake for each participant in the subsample (*n* = 40).

#### Iodine method

This method assumed that iodized salt was the primary source of iodine intake. Therefore, the female's discretionary salt intake was estimated based on the total iodine content of her duplicate diet composite and the iodine concentration of her household salt sample, while also accounting for other dietary iodine sources consumed on the day of assessment [18]. Although fish and seafood products were not consumed in the study area, we observed that dairy products contained iodine due to disinfection practices with iodophor. To estimate discretionary salt intake using the IM, the following steps were undertaken: *1*) the weight of milk (cow or buffalo) consumed by participants was quantified, and the amount of iodine contributed by the consumed milk was quantified using the mean analytical values measured from pooled milk samples per study village; *2*) the amount of iodine contributed by the consumed milk was then subtracted from the total iodine content of the duplicate diet composite; and *3*) the difference between the total iodine and the milk iodine gave the final iodine content of the duplicate diet. Using the iodine concentration of the participant’s salt sample, discretionary salt intake was calculated based on the nonmilk iodine content of the duplicate diet composites.

### Statistical analysis

Background characteristics of study participants were summarized using descriptive statistics. The observed discretionary intake estimates from the WFR and HHSD method that had repeat measurements were adjusted to estimate the distribution of usual intakes using the multiple source method (MSM) [19]. This MSM involved the use of linear regression to estimate the amount of food consumed on a consumption day. Residuals from the model were transformed using a Box-Cox function to normalize the data. The transformed residuals were then used to estimate inter- and intraindividual variance, ensuring that intraindividual variation was removed. After adjusting for intraindividual variation, the intake estimates were back-transformed to the original scale. The final usual intake distribution was estimated by multiplying the probability of consumption by the usual amount consumed. Scatter plots and Spearman correlation coefficients were used to assess the association between the observed discretionary salt estimates measured via the reference method (i.e. WFR) compared with the HHSD, RD, and IM methods. Bland–Altman plots were used to assess the agreement between the observed discretionary salt estimates from the WFR and the HHSD, RD, and IM methods [20]. Statistical analyses were conducted using SAS version 9.4.

## Results

Mean participant age was 35 y, and more than half of the participants were classified as overweight or obese (Table 1). The majority (98%) of participants had a primary education or higher. The median monthly income of the households was 12,000 Indian Rupees (equivalent to ∼ US$144). Refined iodized salt was the primary source of salt used in cooking and seasoning in all households. The majority of households bought 1 kg of refined iodized salt at each purchase. The frequency of salt purchases was biweekly for 21% of households, monthly for 66% of households, and bimonthly or less frequently for 13% of households.

**TABLE 1:** Sociodemographic characteristics and salt utilization and purchasing practices of study participants.

	Mean ± SD or %
Female characteristics (*n* = 100)	
Age (y)	34.5 **±** 6.9
BMI (kg/m^2^) (WHO cut-offs)	26.4 ± 5.5
Underweight < 18.5 kg/m^2^	7.0
Normal 18.5–24.9 kg/m^2^	38.0
Overweight 25.0–29.9 kg/m^2^	31.0
Obese > 30 kg/m^2^	24.0
BMI (kg/m^2^) (Indian cut-offs)	
Underweight < 18.0 kg/m^2^	6.0
Normal 18.0–22.9 kg/m^2^	21.0
Overweight 23.0–24.9 kg/m^2^	18.0
Obese > 25 kg/m^2^	55.0
Education completed	
None	2.0
Middle/secondary	72.0
Diploma/postgraduate	26.0
Marital status	
Married	87.0
Separated/never married	13.0
Household/socioeconomic characteristics	
Monthly income (INR)^1^	12,000 (10,000, 25,000)
Ownership of ration card^2^	67.0
Number of people living in household^3^	5.8 ± 2.1
Salt utilization and purchase practices	
Refined iodized salt the main household salt	100.0
Types of salt used to season food at table (*n* = 47)	
Refined iodized salt	21.0
Pink Himalayan salt	2.0
Black salt	77.0
Salt is shared outside the home	24.0
Primary source of salt purchase	
Corner shops	58.0
Market	40.0
PDS/ration shops	2.0
Quantity of salt per purchase	
0.5 kg	1.0
1 kg	62.0
2 kg and greater	37.0

Values are mean ± SD, *n* (%); *n* = 100, unless otherwise noted.

Abbreviation: PDS, Public Distribution System.

1 Values for average income are the median (IQR) in Indian Rupees (INR).

2 A ration card allows households to buy some household food items at costs subsidized by the government.

3 A household was defined as a group of people who are primarily dependent on an individual person, currently eat from the same pot, and live under the same roof or in the same compound.

**FIGURE 1:**
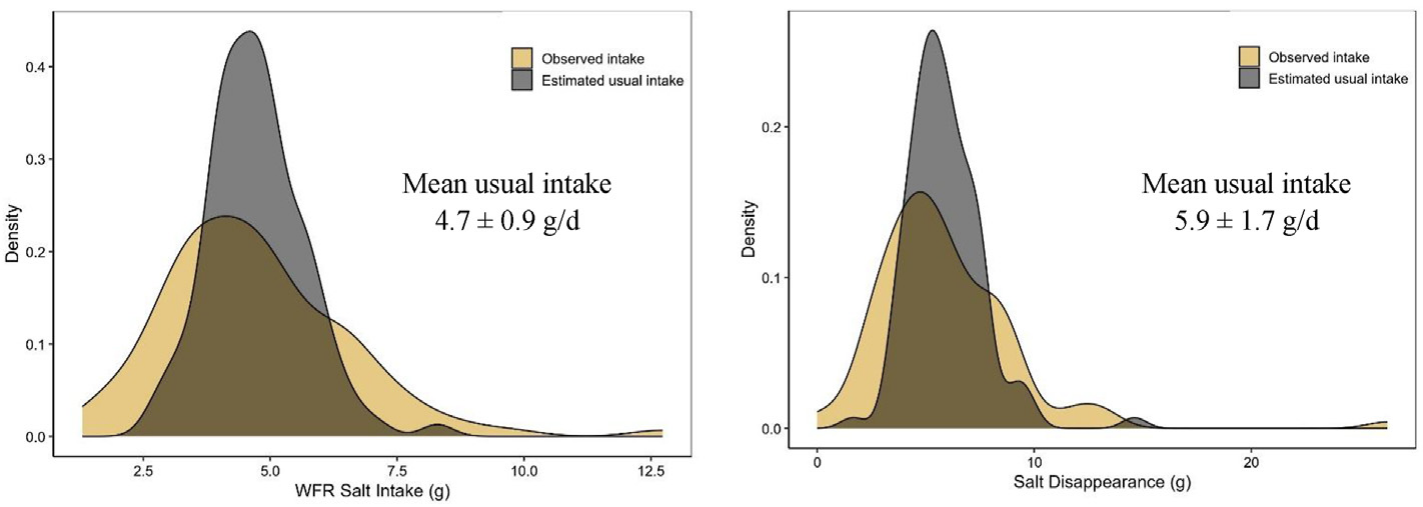
Estimated usual discretionary salt intake using weighed food record and household salt disappearance data. This figure shows the density plots of observed and usual discretionary salt intakes using the estimates from the weighed food record method (reference method) and the household salt disappearance method. The distribution of usual discretionary intakes was estimated using the multiple source method [20]. WFR, weighed food record.

**FIGURE 2:**
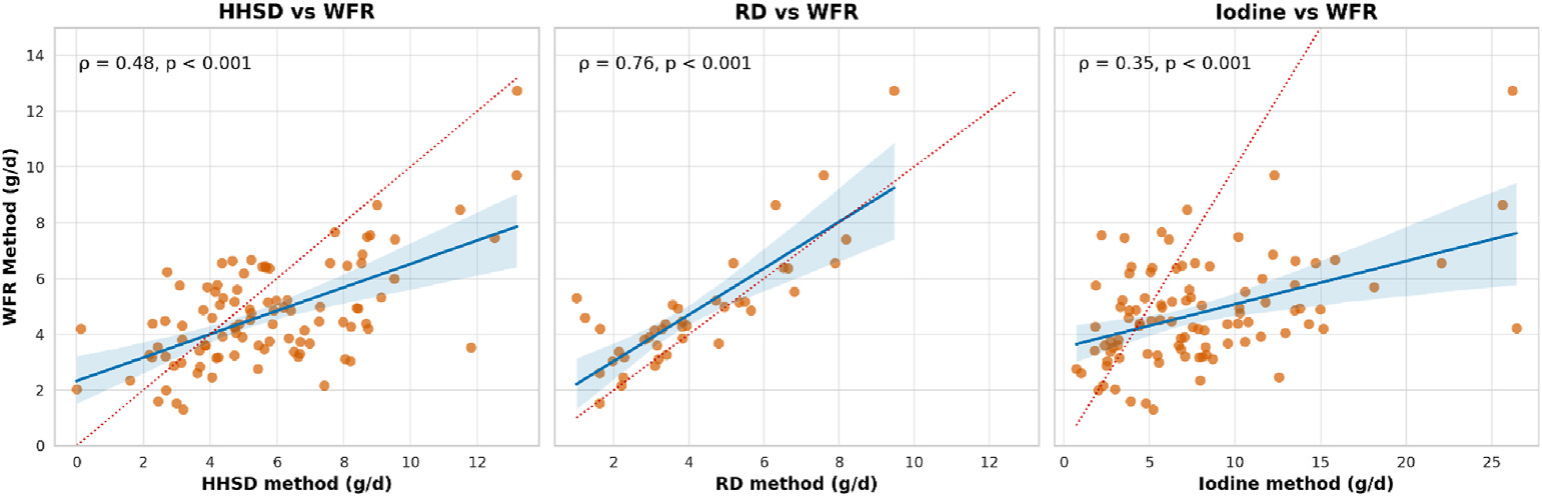
Scatter plots of discretionary salt intake using the weighed food record method vs. the household salt disappearance, replicate diet, and iodine methods. The figure shows the scatter plots and Spearman correlation coefficients between the weighed food record method (reference method), and the household salt disappearance method, the replicate diet, and iodine methods. The replicate method includes data from a subgroup of 40 participants for which duplicate diet replicates were available. Each plot includes a blue regression line with a confidence interval (shaded blue region) and a dotted red reference line representing the line of equality (*y* = *x*). HHSD, household salt disappearance; RD, replicate diet method; WFR, weighed food record.

Mean ± SD discretionary salt intakes according to the WFR, HHSD, RD, and IM methods were 4.7 ± 1.8 g/d, 5.7 ± 2.6 g/d, 4.1 ± 2.1 g/d, and 7.8 ± 5.3g/d, respectively (Table 2). The WFR data were most precise (between-person coefficient of variation (CV) = 39%) whereas the IM data showed the lowest levels of precision (between-person CV = 67%). After adjusting observed discretionary intakes to estimate the distribution of usual salt intakes, the mean ± SD discretionary salt intakes for WFR and HHSD methods were 4.7 ± 0.9 g/d and 5.9 ± 1.7 g/d, respectively (Figure 1). Discretionary salt intake constituted 70.4% ± 17.6% of total salt intake in the subset of 40 participants for whom RDs were collected. Spearman correlation coefficients for estimates of discretionary salt intake obtained from WFR compared with HHSD, RD, and IM methods were 0.48 (*P* < 0.001), 0.76 (*P* < 0.001), and 0.35 (*P* < 0.001), respectively (Figure 2).

**TABLE 2:** Mean intake of discretionary salt among study participants using 4 assessment methods.

Method	*N*	Mean ± SD	CV (%)	Median (IQR)	Min	Max
Weighed food record	100	4.7 ± 1.8	38.9	4.4 (3.3–5.7)	1.3	12.7
Household salt disappearance^1^	99	5.7 ± 2.6	46.4	5.2 (3.9–7.4)	0.0	13.2
Replicate diet method	40	4.1 ± 2.1	50.8	3.6 (2.5–5.4)	1.0	9.5
Iodine method^2^	98	7.8 ± 5.3	67.4	6.9 (3.9–10.3)	0.72	26.4

Abbreviation: CV, coefficient of variation.

1 One outlier of 26.2 g was excluded from this analysis.

2 Two outliers (salt intake of 95 g and 36.2 g) were excluded from this analysis.

**FIGURE 3:**
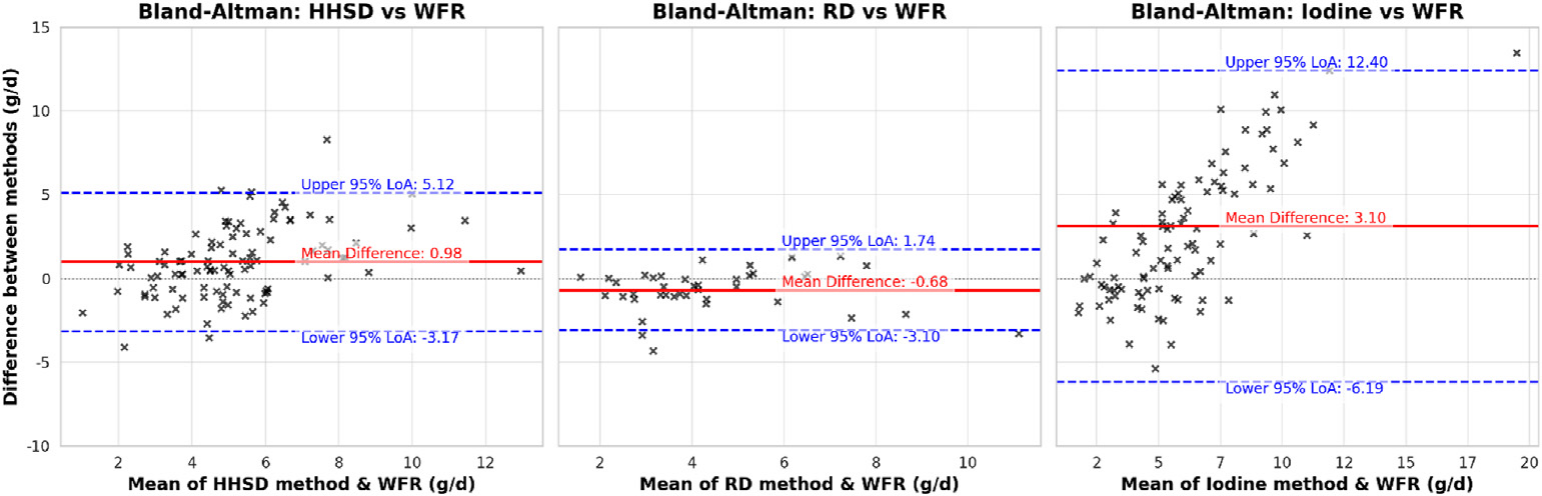
Bland–Altman plots of discretionary salt intake using the weighed food record method (reference method) vs. the household salt disappearance, replicate diet, and iodine methods. The figure shows the Bland–Altman plots for the weighed food record method (reference), the household salt disappearance, the replicate diet, and iodine methods. The *x*-axis is the mean of the reference and comparator methods, and the *y*-axis is the difference between the reference and comparator methods for all 3 plots. The replicate diet method includes data from a subgroup of 40 participants for which duplicate diet replicates were available. Each plot includes a red solid line for the mean difference (bias) and blue dashed lines for the 95% limits of agreement, indicating the range within which most differences fall. HHSD, household salt disappearance; RD, replicate diet method; WFR, weighed food record.

The mean difference ± SD (lower limit of agreement, upper limit of agreement) was lowest between the RD and WFR methods [−0.68 ± 1.25 (−3.18, 1.82)] g/d and it was the only comparison method where the mean difference did not seem to increase with increasing discretionary salt intake (Figure 3). There was also a relatively low mean difference between the HHSD and WFR method [0.98 ± 2.12 (−3.19, 5.23)] g/d (Figure 3). The mean difference was highest, and the limits of agreement were widest between the IM and WFR method [3.10 ± 4.77 (−6.43, 12.63)] g/d (Figure 3).

## Discussion

In this comparative study of 4 methods for the estimation of discretionary salt intake among nonpregnant females of reproductive age in Punjab, India, we found good agreement between the WFR method (reference) and the RD and HHSD methods, but not with the IM method. The mean estimates of discretionary salt intake in the study ranged from 4.1 to 7.8 g/d, depending on the method used for estimation. Our results demonstrated that the RD method had the strongest correlation and best agreement with the WFR method. The highest estimate of mean discretionary salt intake was generated from the IM, which also showed the greatest variability in intake with a coefficient of variation of 67% compared with 39% for the WFR method, a mean difference of 3.10 g/d, and the widest limits of agreement. Given the range of estimates of discretionary salt in the literature, the mean difference between the WFR and IM methods seems greater than would be acceptable for most use cases.

The current study is one of the few studies that have compared methods for assessing discretionary salt intake in a resource-limited field setting and adds to the dearth of literature on the subject. We employed comprehensive and intensive dietary assessment and analytical methods to generate estimates of discretionary salt intake. The use of the WFR method as the reference method represents a strength of the study, as this method is not subject to recall bias and is considered a reference standard method for assessing energy and nutrient intakes. In the literature, the majority of the estimates of discretionary salt intake are generated using 24-h recalls because it is much easier to implement [8]. The collection of duplicate diet composites, which we used to analyze sodium and iodine concentrations, is also an accepted reference standard method for assessing micronutrient intakes. There were, however, some limitations that could have affected the precision of our methods. The crude HHSD method assumes that everyone in the household had the same discretionary salt intake, which is known to be false. A systematic review of total salt intake in India stratified by sex showed that men’s average total salt intake was 9.6 g/d whereas the estimate for females was lower at 6.6 g/d [21]. The precision of the HHSD method could be improved by collecting data on household composition, which would enable salt utilization to be expressed per adult female equivalent [22]. Another weakness of the HHSD method is that there can be days when a household eats only leftover foods or cooks in large batches for an occasion or event, or uses salt for nonconsumption purposes, and these can heavily skew the mean discretionary salt intake values. Repeat measurement days may help address this limitation, and although we repeated the HHSD study in 40 participants, the precision may have improved further with 1 or more repeat assessments in all participants. The IM demonstrated the weakest correlation, highest mean bias, and widest limits of agreement compared with the WFR method. This method had several limitations, which likely contributed to its imprecision and bias. Forty-one percent of households in our study population owned livestock (cow or buffalo) for milk production. Iodine is commonly used as a teat disinfectant and has been found to increase the concentration of iodine in milk through contamination [23]. This informed our decision to collect and pool household milk samples by the community and analyze them for iodine concentrations. Measuring the iodine in milk from the pooled milk samples instead of individual household samples may have also introduced some errors. We were not able to properly account for iodine contributed by processed foods, condiments, and other milk products due to missing iodine content information. Another drawback of our study was the lack of the 24-h recall method, which is the most commonly used to assess discretionary salt in the literature. The reference WFR method is also not without limitations. The presence of the research assistant in the participant’s home could have caused the participant to consciously or unconsciously alter their eating habits, which may have potentially affected their recorded discretionary salt intake on the day of the dietary assessment. However, these limitations were anticipated, and participants were carefully instructed not to change their cooking or eating patterns during the dietary assessment.

The current study was part of formative research and informed the micronutrient fortification levels for a randomized controlled trial of multiply-fortified salt [24]. The discretionary salt intake estimates from the WFR method were used to model micronutrient fortification levels that would significantly reduce the prevalence of inadequate micronutrient intakes while limiting intakes above the tolerable upper intake level among nonpregnant females of reproductive age in this population. Our sample size was small and allowed for the exploration of these 4 methods with varying degrees of implementation ease. The choice of method for future salt fortification interventions or salt reduction efforts will depend on several factors, including technical, material, and financial resources, time available, and scale of the intervention. All methods used in our comparative study require a full day of in-home assessments and are, therefore, quite intensive to implement. However, the least burdensome method to implement is the HHSD method. Although crude, the HHSD method showed good agreement with the WFR method, which provides some assurance that discretionary salt intake estimates from this method may be reasonable for most cases but likely overestimate actual discretionary salt intake. Additionally, the HHSD method is more feasible to implement in resource-limited, field-based settings as it does not require expensive equipment or advanced technical knowledge. Although the RD method agreed best with the WFR method, it required the collection and homogenization of duplicate diet composites and laboratory analysis of sodium, which is demanding from time, labor, and financial perspective, and would not be a practical choice for large-scale field surveys. The IM also relied on several calculations and assumptions, which likely introduced errors and resulted in estimates that were the least precise.

In general, the trend among the limited studies that have compared different methods of estimating discretionary salt intake indicates that the more rigorous the method, the smaller the estimate of discretionary salt intake [25,26]. A study in New Zealand compared discretionary sodium intake estimates among 109 male and female adults using the lithium-marker technique, 24-h dietary recall, and the “subtraction method” a combination of the 24-h urine and 24-h recall estimates [27]. The estimate from the 24-h recall was almost double (994 ± 1385 mg sodium/d) the estimate from the lithium-marker technique (537 ± 642 mg sodium/d) whereas the estimate (675 ± 1545 mg sodium/d) from the “subtraction method” was ∼138 mg more than the lithium-marker technique. Another small study among 9 females in Guatemala produced estimates of discretionary salt intake of 3.9 ± 2.6 g/d for the lithium-marker technique, which was twice and 3 times the values using 24-h recall (7.4 ± 4.9 g/d) and double portion method (12.8 ± 6.5 g/d), respectively [8]. A similar trend was observed in a study among females in East Java, Indonesia where the estimates of discretionary salt from a 24-h salt recall (7.01 ± 2.44 g/d) and HHSD data (6.0 ±1.8 g/d) were also at least twice the estimate from the lithium-marker technique (2.99 ± 1.5 g/d) [9]. The correlation coefficients between the 24-h recall and the lithium-marker method in the above studies ranged between 0.32 and 0.76.

In conclusion, the current study demonstrates that there was good agreement between the WFR and the HHSD and RD methods, but not the IM method. If adequate resources and technical expertise are available, the WFR and RD methods are appropriate options for the estimation of discretionary salt intake in smaller field surveys and/or studies. The RD method requires additional effort beyond the WFR method and does not provide much added value apart from confirming the quality of the WFRs. The HHSD method is more accessible and less resource intensive and would work well for large-scale population-based surveys. However, further research should explore the development and application of population-specific correction factors to produce more accurate estimates of actual discretionary salt intake.

## Author contributions

The authors’ responsibilities were as follows – CMM, KHB, MSM, NFK, RSG, MD, RD: designed and supervised research; YEG, SA, SS, DB, BLS, MJ, JW, JML: conducted research; YEG, CDA: analyzed data; YEG: wrote the first draft manuscript; and all authors: read, edited, and approved the final manuscript.

## Data availability

Data described in the manuscript, code book, and analytic code will be made available on request pending approval by principal investigator.

## Funding

The study was funded by the Bill & Melinda Gates Foundation, grant number (INV-002945).

## Conflict of interest

KHB is a part-time consultant to the BMGF but had no role in the evaluation or funding decision for the current grant. All other authors report no conflicts of interest.

## References

[1] Zimmermann M.B. (2023). The remarkable impact of iodization programmes on global public health. Proc. Nutr. Soc..

[2] Diosady L.L., Mannar M.G., Krishnaswamy K. (2019). Improving the lives of millions through new double fortification of salt technology, Matern. Child Nutr..

[3] Modupe O., Diosady L.L. (2021). Quadruple fortification of salt for the delivery of iron, iodine, folic acid, and vitamin B12 to vulnerable populations. J. Food Eng..

[4] World Health Organization (2007).

[5] (2023). WHO global report on sodium intake reduction.

[6] Titze J. (2017). Estimating salt intake in humans: not so easy. Am. J. Clin Nutr..

[7] Leclercq C., Avalle V., Ranaldi L., Toti E., Ferro-Luzzi A. (1990). Simplifying the lithium-marker technique used to assess the dietary intake of discretionary sodium in population studies. Clin. Sci. (Lond)..

[8] Bhat S., Marklund M., Henry M.E., Appel L.J., Croft K.D., Neal B. (2020). A systematic review of the sources of dietary salt around the world. Adv. Nutr..

[9] Goh Y.E., Manger M.S., Duggal M., Das R., Saklani S., Agarwal S. (2023). Women in selected communities of Punjab, India have a high prevalence of iron, zinc, vitamin B12, and folate deficiencies: implications for a multiply-fortified salt intervention. Nutrients.

[10] (2022). District SAS Nagar. https://sasnagar.nic.in/.

[11] International Institute for Population Sciences (IIPS) and ICF (2021).

[12] Food and Agriculture Organization (2018).

[13] AACC (2000).

[14] Todorov T.I., Gray P.J. (2016). Analysis of iodine in food samples by inductively coupled plasma-mass spectrometry. Food Addit. Contam. Part A Chem. Anal. Control Expo. Risk Assess.

[15] Hammer D., Andrey D. (2008). Comparison of ion-selective electrode and inductively coupled plasma-mass spectrometry to determine iodine in milk-based nutritional products. J. AOAC Int..

[16] Chan K., Gallant J., Leemaqz S., Baldwin D.A., Borath M., Kroeun H. (2021). Assessment of salt intake to consider salt as a fortification vehicle for thiamine in Cambodia. Ann. N. Y. Acad. Sci..

[17] Agbemafle I., Woldeyohannes M., Tessema M., Fereja M., Arnold C.D., Banjaw B.T. (2025). Assessment of women's discretionary salt intake and household salt utilization in preparation for a salt fortification trial in Oromia region, Ethiopia, Matern. Child Nutr..

[18] Iacone R., Iaccarino Idelson P., Russo O., Donfrancesco C., Krogh V., Sieri S. (2021). Iodine intake from food and iodized salt as related to dietary salt consumption in the Italian adult general population. Nutrients.

[19] Bland J.M., Altman D.G. (1986). Statistical methods for assessing agreement between two methods of clinical measurement. Lancet.

[20] Harttig U., Haubrock J., Knüppel S., Boeing H., Consortium EFCOVAL (2011). The MSM program: web-based statistics package for estimating usual dietary intake using the multiple source method. Eur. J. Clin. Nutr..

[21] Johnson C., Praveen D., Pope A., Raj T.S., Pillai R.N., Land M.A. (2017). Mean population salt consumption in India: a systematic review. J. Hypertens..

[22] Weisell R., Dop M.C. (2012). The adult male equivalent concept and its application to Household Consumption and Expenditures Surveys (HCES). Food Nutr. Bull..

[23] O’Brien B., Gleeson D., Jordan K. (2013). Iodine concentrations in milk. Irish J. Agric. Food Res..

[24] McDonald C.M., Brown K.H., Goh Y.E., Manger M.S., Arnold C.D., Krebs N.F. (2022). Quintuply-fortified salt for the improvement of micronutrient status among women of reproductive age and preschool-aged children in Punjab, India: protocol for a randomized, controlled, community-based trial. BMC Nutr.

[25] Melse-Boonstra A., Rexwinkel H., Bulux J., Solomons N.W., West C.E. (1999). Comparison of three methods for estimating daily individual discretionary salt intake: 24 hour recall, duplicate-portion method, and urinary lithium-labelled household salt excretion. Eur. J. Clin. Nutr..

[26] Mustafa A., Muslimatun S., Untoro J., Lan M.C., Kristianto Y. (2006). Determination of discretionary salt intake in an iodine deficient area of East Java-Indonesia using three different methods, Asia Pac. J. Clin. Nutr..

[27] McLean R.M., Wang N.X., Cameron C., Skeaff S. (2023). Measuring sodium from discretionary salt: comparison of methods. Nutrients.

